# Transvenous occlusion of incompetent pelvic veins to treat chronic pelvic pain in women: A randomised controlled trial

**DOI:** 10.1111/1471-0528.17512

**Published:** 2023-04-24

**Authors:** Vivak Hansrani, David Riding, Mourad W. Seif, Ann-Louise Caress, Katherine Payne, Jonathan Ghosh, Charles N. McCollum

**Affiliations:** 1 Division of Cardiovascular Sciences, School of Medical Sciences, University of Manchester, Manchester, UK; 2 Manchester Vascular Centre, Manchester University NHS Foundation Trust, Manchester, UK; 3 St. Mary’s Hospital, Manchester University NHS Foundation Trust, Manchester, UK; 4 Health Services Research, Department of Nursing and Midwifery, School of Human and Health Sciences, University of Huddersfield, Huddersfield, UK; 5 Health Economics, Institute of Population Health, University of Manchester, Manchester, UK

**Keywords:** pelvic pain, randomised controlled trials

## Abstract

**Objective:**

To investigate the effectiveness of transvenous occlusion of incompetent pelvic veins in women presenting with chronic pelvic pain (CPP) in improving symptoms and quality of life

**Design:**

Patient-blinded randomised controlled trial with objective outcome measures. Results were analysed on an intention-to-treat basis

**Setting:**

Gynaecology and Vascular Surgery Services of two teaching hospitals in northwest England.

**Population:**

Sixty women aged 18–54 years presenting with CPP after exclusion of other pathology, and who were found to have pelvic vein incompetence

**Methods:**

Participants were randomised and assigned to contrast venography alone or contrast venography plus transvenous occlusion of the incompetent pelvic veins

**Main outcome measure:**

The primary outcome was change in pain score measured using the short-form McGill Pain Score (SF-MPQ) and the Visual Analogue Score (VAS) recorded at 12 months post-randomisation. Secondary outcomes include quality of life using the EQ-5D instrument, symptomatic improvement and procedure-related complications

**Results:**

Sixty participants were randomised to transvenous occlusion of incompetent pelvic veins or venography only. At 12 months, median pain scored 2 (3–10) in the intervention group versus 9 (5–22) in controls (*p* = 0.016). Pain on the VAS scored 15 (0–3) versus 53 (20–71), respectively (*p* = 0.002). Median EQ-5D improved after intervention from 0.79 (0.74–0.84) to 0.84 (0.79–1.00; *p* = 0.008) over 12 months. No major complications were reported

**Conclusion:**

Transvenous occlusion of pelvic vein incompetence reduced pain scores, improved quality of life and diminished symptom burden with no major reported complications

**Trial registration:**

ISRCTN 15091500.

## 1 Introduction

Chronic pelvic pain (CPP) is defined as infra-umbilical and/ or pelvic pain that persists for longer than 6 months and is not associated with intercourse, pregnancy or the menstrual cycle.^1,2^ It is extremely common throughout the western world, affecting 3.8% of all women in the western world annually, a similar incidence to asthma and back pain.^2^ CPP accounts for over 30% of all gynaecology outpatient appointments and is the most frequent indication for surgery in women, accounting for 40% of laparoscopies and 12% of hysterectomies. No cause can be identified in more than 55% of cases.^3,4^ CPP often leads to impaired quality of life, social isolation, marital discord and frequent absence from, or loss of, work or employment.^5^ Women with CPP frequently become disheartened and disengaged with healthcare services.^6^

Pelvic vein incompetence (PVI) was first described by Taylor in 1949 and has been suggested as a possible cause of pelvic pain.^7^ Observational data suggest that it can affect 15–20% of women, but it remains poorly understood.^8,9^ The most common theory is that symptoms arise from persistent retrograde flow in the ovarian and internal iliac veins leading to venous hypertension in the pelvic venous plexus. There is presently no guidance on the management of PVI from the United Kingdom Royal College of Obstetrics and Gynaecology (RCOG) or the National Institute for Health and Care Excellence. The equivalent condition in men, testicular varicoceles, is also caused by gonadal vein incompetence and is treated in the UK by the National Health Service (NHS). In women, various treatment options have been suggested including hormonal therapy, surgical venous ligation and even hysterectomy. Attempts to eliminate pelvic hypertension by occlusion of incompetent veins using coils, glue, sclerosants or a combination via percutaneous access, to eliminate venous reflux are the most popular forms of intervention in the private sector and are not widely available in the NHS. These treatments have not been evaluated in randomised control trials but are still regularly performed in private payment and insurance-based healthcare systems.^10^

To address the paucity of evidence, this randomised control trial aimed to investigate the effectiveness of transvenous occlusion of incompetent pelvic veins in women with CPP. The impact on pain, quality of life and complications were also evaluated.

## 2 Methods

### 2.1 Study design

Between June 2016 and February 2020, patients were enrolled into a randomised, single-blinded parallel clinical trial conducted at two large university teaching hospitals in northwest England. Local ethics committee approval was obtained (NRES reference NRES 15/NW/0360). All potential participants were provided with study information leaflets and written consent was obtained from those who agreed to take part. A trial steering committee and an independent data monitoring committee provided study oversight.

### 2.2 Participants

Women aged 18–54 years (inclusive) with unexplained CPP were eligible. All patients were referred to the study by their gynaecologist. Each patient’s gynaecologist was required to confirm that their patient fulfilled the RCOG criteria for CPP and that no further investigations were planned. This was the point at which they would normally be discharged back to their primary care physician or to a chronic pain service. We anticipated that the patient's gynaecologist would have under-taken a detailed assessment, which often included pelvic ultrasound, cross-sectional imaging and laparoscopy to identify a cause for the CPP symptoms. Specific investigations were not mandated before a patient could be referred to the study.

Women were excluded if they were pregnant or within 12 months of pregnancy, found to have alternative pathologies that may cause CPP, had undergone previous hysterectomy, or were unable to give informed consent.

Women meeting the inclusion criteria underwent a transvaginal Duplex ultrasound (TVDU) to detect or exclude PVI and pelvic varices. PVI was defined, by a Delphi Consensus Study of the British Society of Interventional Radiology members, as sustained reflux greater than 0.5 seconds in the ovarian and/or internal iliac veins generated by Valsalva’s manoeuvre.^11^

### 2.3 Randomisation and blinding

A computer-generated variable block randomisation was derived by the Medical Statistics Team at the Royal College of Surgeons of England’s Northwest Clinical Trials Unit. Participants were assigned in equal numbers (1:1) to either reflux venography plus coil embolisation, or to reflux venography alone. Each participant’s gynaecologist was informed of the outcome, but the patient was blinded to their allocation.

### 2.4 Screening

A standardised protocol for the detection of PVI using TVDU was performed.^12^ The transvaginal duplex probe was introduced in a supine position. The presence of reflux in the ovarian, internal iliac and para-uterine veins on both sides was assessed during Valsalva’s manoeuvre. The presence of reflux was then assessed in the semi-erect position with the patient sat on the edge of the examination couch. For this study, sustained reflux lasting longer than 0.7 seconds was defined as PVI. The results of TVDU were reported to all participants and their physician with an explanation of the significance of the findings. Lower limb venous reflux was not assessed during this visit.

### 2.5 Intervention

Transjugular venography was performed in an interventional radiology suite under local anaesthesia. Using ultrasound guidance, the internal jugular vein was accessed using the Seldinger technique. For each participant, selective catheterisation was performed and the presence of venous incompetence was assessed with Valsalva’s manoeuvre. Evidence of reflux, venous engorgement, pelvic varices or communication across the midline or thigh and/ or vulvovaginal varices was noted. Once PVI and/or pelvic varices were identified, a web-based randomisation tool was used and the outcome was reported to the interventional radiologist, who proceeded to either perform coil embolisation of the incompetent veins, or end the procedure. In an effort to conceal allocation, patients were given headphones and were prevented from viewing fluoroscopy during the procedure. Sedation and opiate analgesia were offered pro re nata.

In those patients randomised to treatment, metallic coils were deployed at the discretion of the interventional radiologist. In the internal iliac veins, coil embolisation was only performed in incompetent second-order branch veins and was not pursued into third-order or subsequent branches into the buttock or thigh. After embolisation, venography was performed to confirm occlusion of the treated veins and cessation of reflux. Patients were discharged home on the day of the procedure and were advised to self-medicate with paracetamol and ibuprofen as required. Oral medication such analgesic, hormonal, or contraceptive therapies were not stopped during the trial period.

### 2.6 Outcomes

The primary outcome measure was Short Form-McGill Pain Questionnaire (SF-MPQ) scored at 12 months post-randomisation. The SF-MPQ was selected because a disease-specific CPP validated outcome measure did not exist, and it is one of the most frequently used outcome tools in CPP studies.^13^ The SF-MPQ encompasses three parameters: pain rating index (maximum 45), current or present pain visual analogue scale (VAS; 0–100), and current pain intensity (range 0–5). Of these, pain rating index was chosen as the primary parameter. All scores were completed at 1 week, followed by 3 and 6 months.

Secondary outcome measures included the EQ-5D-3L (EuroQol five dimension, three levels).^14^ Published UK social preference weightings were used to transform EQ-5D-3L scores into a measure of health-related quality of life. A symptom questionnaire designed by our patient and public involvement (PPI) group to collect changes in symptoms using the VAS. All outcome measures were completed online.

All participants were contacted by telephone after 1 week to check for complications and the use of post-procedure analgesia.

### 2.7 Power calculation

The smallest difference in the SF-MPQ pain rating index considered in the literature to be clinically important is reported to be five points (max 45).^13,15,16^ As such, we needed 36 individuals in each group to detect this five-point difference at 80% power using an unpaired *t* test at the conventional 5% significance level, assuming the published standard deviation of 7.4. This sample size was inflated by 10% to incorporate potential prognostic factors in a covariance regression model. A further 10% was added to compensate for potential loss to follow up. The total number of women in each group was then rounded up to 50 (100 in total).

### 2.8 Statistical analysis

The Northwest Royal College of Surgeons of England’s North West Clinical Trials Unit was responsible for managing the trial database. The primary analysis compared changes in pain scores (pain rating index and VAS) from baseline to 12 months post-randomisation, using analysis of covariance (ANCOVA) with adjustment for baseline values. The secondary analysis included comparison of changes in outcome from baseline to follow-up time points at 1 week, and then 3, 6 and 12 months post-randomisation. Similarly, changes in quality of life from baseline to each follow-up time point were compared using ANCOVA. Longitudinal regression analysis using generalised estimating equations compared changes in these scores in the two treatment groups throughout the follow-up period. All analyses were conducted on the intention-to-treat basis, using the conventional 5% significance level. R software (version 4.1.0) was used to compute the data.

### 2.9 Patient and public involvement

Our PPI group included five women with CPP and PVI, of whom three women had been treated for PVI with coil embolisation. The Chair of the regional charitable organisation Pelvic Pain Support Network was also a member of our PPI group, and a co-applicant during the development of this study. The group helped design the study treatment pathways and in the absence of a validated CPP score, chose the SF-MPQ as the primary outcome measure. They pilot-tested our questionnaires, ensuring that they were clearly written without medical terminology.

## 3 Results

### 3.1 Study cohort

**Figure 1:**
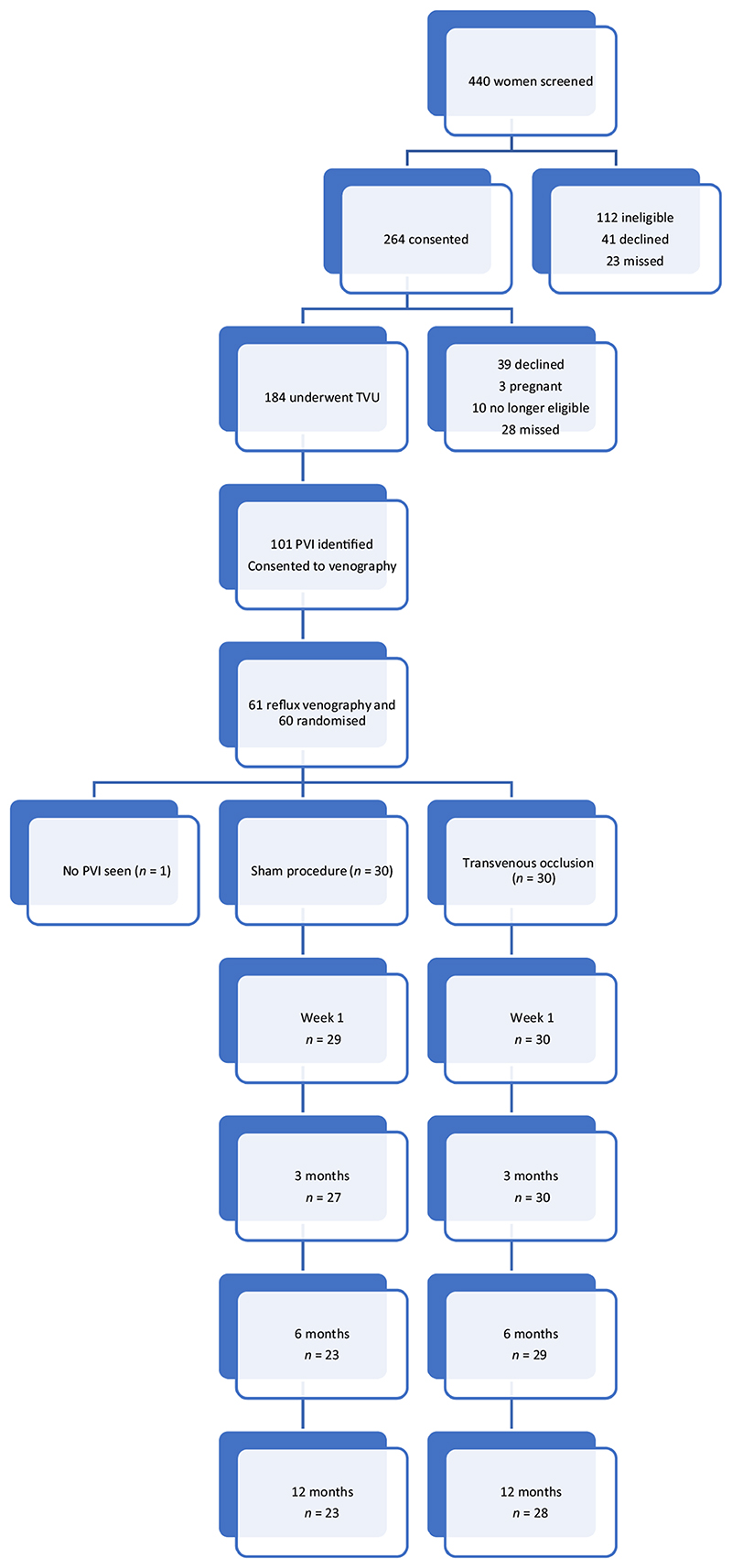
Consort flow diagram.

In total, 440 women with the diagnosis of CPP completed a screening questionnaire to assess eligibility to the study. A total of 264 women consented to take part in the study. Of these 264 women, 184 (70%) underwent a screening TVDU to screen for PVI. Thirty percent of women did not undergo a TVDU (Figure 1). A total of 101 of 184 (55%) women had PVI identified by TVDU and entered the study.

The coronavirus disease 2019 (COVID-19) pandemic resulted in the suspension of the trial in February 2020. Participants were advised not to attend the hospital trial sites, research staff were redeployed and access to the interventional radiology suite was halted. In 2021, with no improvement in outlook for the pandemic, the increased risk of hospital attendance and the continued lack of access to the interventional suite, the trial steering committee and sponsor made the joint decision to stop the trial. By this time, 60 of 101 (59%) patients had undergone their interventional procedure and had been randomised to either coil occlusion or venography alone.

In total, nine (15%) participants were lost to follow up at 12 months (seven controls and two from the intervention arm).

Demographic characteristics were similar in both arms (Table 1). Mean ± standard deviation age was 39 ± 7 years in controls and 40 ± 8 years in the intervention arm. Gravida, parity, smoking history and body mass index were equally distributed. The presence of significant other cardiovascular, respiratory or renal comorbidities was not encountered in this group.

### 3.2 Primary outcome

The SF-MPQ consists of three parameters: pain rating index (maximum 45), current or present pain VAS (0–100) and current pain intensity (range 0–5). Participants randomised to coil embolisation had a progressive reduction in their pain rating index, with median (interquartile range [IQR]) of 14 (IQR 7–22) at baseline reducing to 3 (IQR 0–10) at 12 months. In comparison, the baseline pain rating index for controls at baseline was 15 (IQR 11–21) reducing to 9 (IQR 5–22) at 12 months. There was a statistically significant difference (*p* = 0.016) in the change in pain rating index between the two groups at 12 months, with median reduction of seven in the coil embolisation group and four in control group (Table 2).

The change in pain SF-MPQ VAS, scored between 0 and 100 at 12 months was also significantly different between groups (*p* = 0.002). Median VAS in the coil embolisation arm fell from baseline 43 (IQR 25–74) to 15 (IQR 0–30) at 12 months, with median score reduction of 25. In comparison, the control baseline score of 53 (IQR 41–75) was un-changed at 12 months 53 (IQR 20–71).

**Figure 2:**
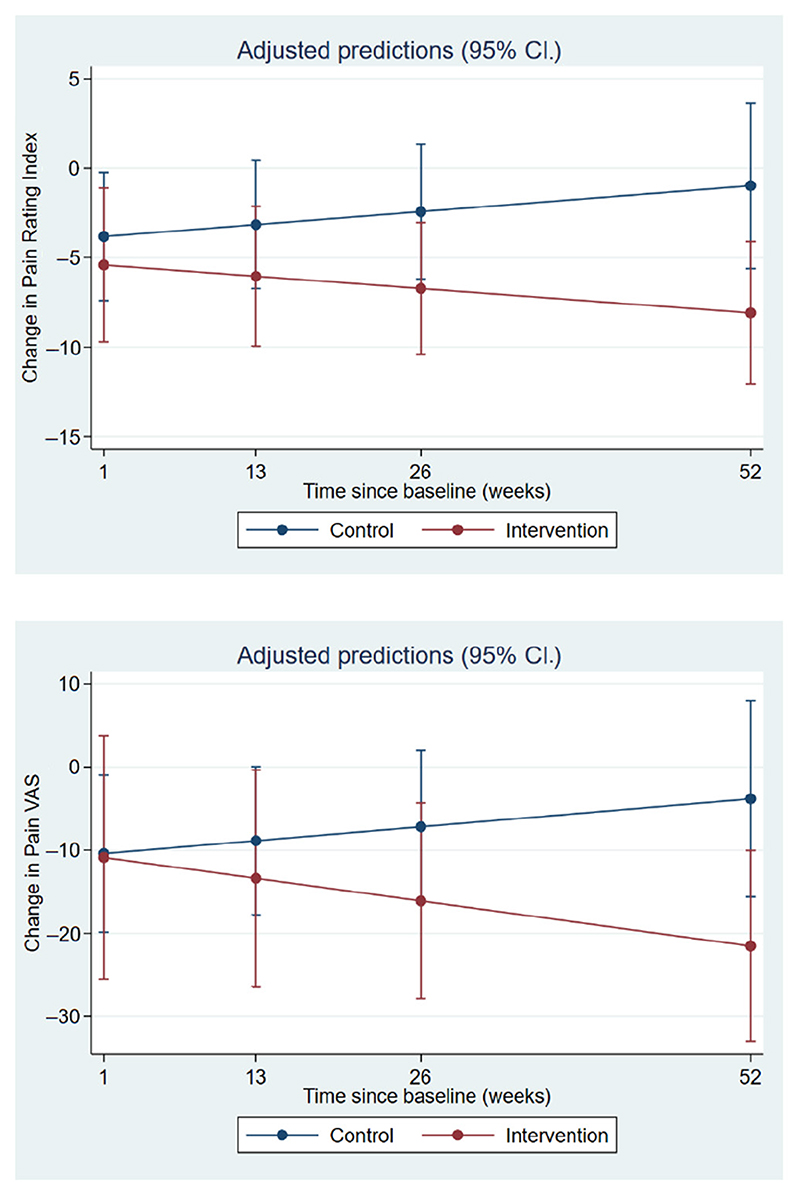
Marginal estimates of changes in pain rating index and visual analogue scale (VAS) at follow up.

Longitudinal analysis found significant differences between the groups in change in pain rating index (*p* = 0.034) and VAS (*p* = 0.036) over the follow-up period, as identified by the treatment*time interaction (Figure 2). The estimated marginal means at each follow-up time point are presented in Table 3.

‘Current’ pain intensity was the third parameter of the SF-MPQ and ranged from 0 to 5 (where 0 = ‘no pain’, 1 = ‘mild’, 2 = ‘discomforting’, 3 = ‘distressing’, 4 = ‘horrible’, 5 = ‘excruciating’). From baseline, a progressive increase was seen in the number of participants selecting ‘no pain’ after coil embolisation. At 12 months, the proportion of patients reporting ‘no pain’ was significantly different between the groups (*p* = 0.005).

### 3.3 Secondary outcomes

There was a significant difference between groups in change in EQ-5D time trade off at 6 and 12 months, adjusting for baseline score (*p* = 0.08) (Table 3). At baseline, median EQ-5D time trade off was 0.77 (IQR 0.68–0.84) in controls compared with 0.79 (IQR 0.74–0.84) in the intervention arm. In the intervention arm, EQ-5D-VAS score was 73 (IQR 51–80) at baseline and increased to 80 (IQR 74–90) at 12 months. In comparison, the control arm EQ-5D-VAS was 75 (IQR 55–83) baseline and decreased to 70 (IQR 55–91) at 12 months. There was a significant difference between groups in change in EQ-5D-VAS, adjusting for baseline score, at 12 months (*p* = 0.01).

### 3.4 Pain characteristics

VAS scores for premenstrual pain, menstrual pain and dyspareunia were also completed. Premenstrual pain VAS in the intervention group fell from median 50 (IQR 25–67) to 25 (IQR 0–49.5) at 12 months compared with 72.5 (IQR 50–75) to 43 (IQR 30–60.5) in controls. Menstrual pain VAS in the intervention group fell from 50 (IQR 25–75) at baseline to 39 (IQR 0–50.5) at the end of 12 months, with the control group reducing from 57.5 (IQR 50–75) to 50 (IQR 41–73.5) (*p* = 0.105). Dyspareunia VAS in the intervention group fell from 37.5 (IQR 0–75) at baseline to 0 (IQR 0–37) at 12 months, compared with 45 (IQR 25–69.2) to 34 (IQR 18.8–54.8) in the control group. There was no significant difference between the groups in either change in premenstrual (*p* = 0.102), menstrual (*p* = 0.105) or dyspareunia VAS (*p* = 0.08) at 12 months.

### 3.5 Technical success and complications

In total, 61 patients underwent catheter venography. One patient was found to have no pelvic reflux or varices, so was not randomised into the study. Of the remaining 60 patients, one (2%) had incomplete coil embolisation due to excessive vasospasm impeding vessel cannulation. The procedure was not re-attempted. The remaining 59 (98%) patients under-went successful coil embolisation. No complications requiring hospital admission were reported. No patients required any further interventional procedures.

## 4 Discussion

### 4.1 Main findings

This single-centre, pragmatic randomised control trial showed that transvenous occlusion of incompetent pelvic veins resulted in reduced pain scores, improved quality of life and reduced symptom burden without complications. These improvements were seen at 12 months after intervention.

Transvenous occlusion reduced both pain rating and intensity. Furthermore, in some patients, the pain burden was eradicated as an increasing number of treated women reported ‘no pain’. This suggests a strong causal relationship, which must be investigated further. The improvement in symptom severity also translated into marked improvements in EQ-5D-3L score. Numerical but non-significant reductions were seen in premenstrual and menstrual pain as well as in dyspareunia.

### 4.2 Strengths and limitations

Women recruited into this study are a good representation of women consulting gynaecologists with CPP in the United Kingdom. All participants had been evaluated by their gynaecologist to exclude extravenous pelvic pathology. At this point, these women would have typically been referred back to their primary care physician. The technical aspects of performing the coil embolisation procedure were left to the discretion of the consultant interventional radiologist. This ensured that technical success and complication rates reflected ‘real-world’ practice. All patients were blinded to their allocation until 12 months follow up had been completed. At the end of follow up, no patient could reliably state to which arm they had been randomised.

Unfortunately, this study was stopped early by the evolving COVID-19 pandemic. Initial planned recruitment was 100 patients. However, a five-point difference in the SF-MPQ was the basis of our power calculation, and this was achieved at the 12-month analysis. Therefore, recruiting to target was unlikely to change this result and the risk of participants coming to harm from COVID-19 was thought to be more significant. Recruitment to the study did take longer than anticipated. Attrition between consenting to the study and attending for the transvaginal ultrasound scan was also higher than expected, with a large proportion failing to attend their ultrasound appointment. Those patients who were confirmed to have PVI on TVDU had long waits before undergoing the intervention procedure as these were procedures added to significant existing radiology demand. Cases with higher clinical need were frequently prioritised over the scheduling of research procedures.

Follow up was limited to 12 months. Longer-term data on pain symptoms and anatomical changes to the pelvic venous system would determine whether coil embolisation affects the natural history of PVI-induced CPP in the long term, and whether patients would need repeated procedures to keep symptoms under control. Follow-up questionnaires were completed online, which helped to keep patients engaged and was easier to complete.

Patients were referred to the study by their treating gynaecologist. It is anticipated that these women would have undergone a number of investigations to identify a cause for their symptoms such as computed tomography, magnetic resonance imaging or diagnostic laparoscopy, but such investigations were not pre-specified to have been undertaken. A limitation of this is the possibility of missing underlying pathology in recruited women.

No attempts were made to gauge technical success or reoccurrence over the follow-up period. Further studies should include interval repeat duplex to assess the success of the procedure. It may be a distinct possibility that women would require more than one embolisation procedure to treat all the incompetent veins.

### 4.3 Interpretation

This is the first randomised control trial of coil embolisation of PVI to provide evidence of a treatment benefit in women with CPP without conferment of significant complications. The trial shows that women receiving coil embolisation can achieve ‘no pain’ and experience an improved quality of life after the intervention. This study highlights the need for a multidisciplinary approach to the management of CPP, and we argue that PVI should be considered as a differential diagnosis, stimulating early referral for investigation.

Women with CPP are significant users of healthcare resources.^17^ They have frequent visits to their general practitioner, attend hospital appointments, have repeated hospital admissions and undergo repeated diagnostic tests and surgery. Economic modelling derived from this trial will be published separately and will assist stakeholders in formulating evidence-based and cost-effective treatment strategies for both PVI and CPP. This evidence should encourage policy-makers to review the lack of guidance and formulate advice to aid clinicians in managing this common condition.

Further studies of PVI and pain symptoms would benefit from longer-term follow up to determine whether the effects of treatment persist. Long-term symptom reoccurrence data would be supported by repeated interval imaging of the treated veins by ultrasound to demonstrate whether the presence of reoccurring pain symptoms coincide with vessel recanalisation or the development of further incompetence or varices. Women with PVI are also associated with more frequent lower limb varicose veins (both primary and recurrent). Future research should study the impact of coil embolisation on lower limb reflux with vein-specific quality of life measures.

## 5 Conclusion

This single-centre, pragmatic randomised study showed that transvenous occlusion of incompetent pelvic veins resulted in reduced pain scores, an improved quality of life and reduced symptom burden with no major complications. These improvements were confirmed at 12 months post intervention.

## Figures and Tables

**Table 1:** Baseline characteristics.

		Controls (*n* = 30)		Embolisation (*n* = 30)	
Age (years)	Mean ±SD	38.5	±6.6	40.1	±8.3
Body mass index (kg/m^2^)	Median (IQR)	23.5	(20.3–27.7)	23.3	(22.1–25.9)
Gravida	Median (IQR)	3	(2–4)	2.5	(2, 3)
Parity	Median (IQR)	2	(0–4)	2	(0–4)
Smoking status
Never smoked	*n* (%)	16	(53.3%)	15	(50.0%)
Ex-smoker		10	(33.3%)	5	(16.7%)
Current smoker		4	(3.3%)	10	(33.3%)

**Table 2:** Primary outcome—short form McGill pain questionnaire scores.

Domain	Time from randomisation	Controls (*n* = 30)		Embolisation (*n* = 30)		*p* value
PRI, median (IQR)	0 days	15	(11–21)	14	(7–22)	–
	1 week	13	(6–17)	5	(3–13)	0.119
	3 months	8	(5–22)	6	(3–12)	0.63
	6 months	11	(6–22)	8	(1–13)	0.196
	12 months	9	(5–22)	3	(0–10)	0.016
VAS, median (IQR)	0 days	53	(41–75)	43	(25–74)	–
	1 week	45	(25–70)	40	(10–60)	0.28
	3 months	30	(16–56)	18	(5–48)	0.23
	6 months	50	(24–73)	38	(4–72)	0.291
	12 months	53	(20–71)	15	(0–30)	0.002

*Note*: PRI: the higher the score, the higher the pain intensity. VAS: patients were invited to mark the scale from ‘no pain (0)’ to ‘worst possible pain (100)’. Abbreviations: IQR, interquartile range; PRI, Pain rating index; VAS, visual analogue scale.

**Table 3:** Secondary outcomes—EQ-5D quality of life scores.

Domain	Time from randomisation	Controls (*n* = 30)		Embolisation (*n* =30)		*p* value
TTO, median (IQR)	0 days	0.770	(0.68–0.84)	0.790	(0.74–0.84)	–
	1 week	0.795	(0.70–0.84)	0.768	(0.74–1.0)	0.167
	3 months	0.782	(0.72–0.84)	0.837	(0.72–0.87)	0.646
	6 months	0.795	(0.68–0.84)	0.837	(0.79–1.0)	0.008
	12 months	0.837	(0.73–0.84)	0.837	(0.80–1.0)	0.008
VAS, median (IQR)	0 days	75	(55–83)	73	(51–80)	–
	1 week	71	(44–93)	78	(46–96)	0.903
	3 months	71	(35–97)	82	(50–94)	0.196
	6 months	78	(38–87)	80	(57–89)	0.182
	12 months	70	(55–91)	80	(74–90)	0.01

Abbreviations: IQR, interquartile range; TTO, time trade off; VAS, visual analogue score.

## Data Availability

The data that support the findings of this study are available on request from the corresponding author. The data are not publicly available due to privacy or ethical restrictions.
